# HLA diversity and signatures of selection in the Maniq, a nomadic hunter-gatherer population in Southern Thailand

**DOI:** 10.1007/s00251-025-01380-0

**Published:** 2025-06-09

**Authors:** Helmut Schaschl, Tobias Herzog, Victoria Oberreiter, Wibhu Kutanan, Mattias Jakobsson, Maximilian Larena

**Affiliations:** 1 Department of Evolutionary Anthropology, Faculty of Life Sciences, University of Vienna, Djerassiplatz 1, 1030 Vienna, Austria; 2 Human Evolution and Archeological Sciences (HEAS), University of Vienna, Djerassiplatz 1, 1030 Vienna, Austria; 3 Konrad Lorenz Institute of Ethology, University of Veterinary Medicine Vienna, Savoyenstraße 1 A, 1160 Vienna, Austria; 4 Department of Biology, Faculty of Science, Naresuan University, Phitsanulok, 65000 Thailand; 5 Human Evolution, Department of Organismal Biology, Uppsala University, Norbyvägen 18 C, 75236 Uppsala, Sweden

**Keywords:** Maniq people, Hunter-gatherer, HLA diversity, Positive selection, Balancing selection

## Abstract

The Maniq, a small and isolated nomadic hunter-gatherer population from the rainforests of Southern Thailand, offer a unique context for investigating how demographic history, genetic drift, and pathogen-driven selection shape human leucocyte antigen (HLA) diversity. Using high-coverage whole-genome data from 21 individuals (12 unrelated), we genotyped HLA alleles with HLA-HD and T1K, identifying 32 alleles in classical and 14 in non-classical HLA genes. Although overall HLA diversity was comparatively low, a few alleles at each locus occurred at high frequency, mirroring patterns observed in other small, isolated populations. Principal-component analysis clustered the Maniq with other Austroasiatic-speaking Semang hunter-gatherers (Jehai, Kintaq) on the Malay Peninsula and, intriguingly, with the Austronesian-speaking Tao of Taiwan, indicating shared immunogenetic features across linguistic boundaries. Despite reduced diversity, multiple loci bore signatures of both long-term balancing and recent positive selection. Several SNPs under selection were in complete linkage disequilibrium with eQTLs known to influence responses to hepatitis B virus (*HBV*) and other pathogens, suggesting that pathogen-driven pressure—in particular *HBV*—may have contributed to recent HLA evolution in the Maniq. These findings provide critical insights into how demographic constraints and pathogen landscapes converge to shape HLA diversity and evolution. In light of increasing infectious disease burdens in indigenous communities, our results underscore the importance of studying small, isolated populations to better understand the adaptive significance of HLA genes.

**Supplementary Information:**

The online version contains supplementary material available at 10.1007/s00251-025-01380-0.

## Introduction

The immunogenetic diversity of indigenous populations remains poorly understood despite its critical importance in uncovering how human immune systems have adapted to different lifestyles and diverse environmental pressures. This gap in understanding is particularly significant for isolated and small hunter-gatherer populations, whose unique evolutionary trajectories can offer key insights into the dynamics of immune function and genetic adaptation. The Maniq people, residing in the rainforests of southern Thailand, are one of the few remaining nomadic hunter-gatherer groups in Southeast Asia (Kricheff and Lukas 2015). We estimate that there are only about 350 Maniq individuals who still pursue a nomadic hunter-gatherer lifestyle (Göllner et al. 2022). Their genetic isolation and traditional lifestyle, combined with exposure to diverse pathogens in their environment, provide a unique context to explore how natural selection has shaped their immune-related genetic diversity.

The human leukocyte antigen (HLA) system is a fundamental component of the immune response, known for its extreme polymorphism, which is crucial for recognizing and presenting antigens to T cells. The HLA genes are located within the major histocompatibility complex (MHC) on chromosome 6 and include both classical, highly polymorphic class Ia and class IIa genes, as well as the limited polymorphic non-classical HLA class Ib and class IIb genes. Due to its extreme diversity of functionally different HLA alleles, the human MHC region has become one of the most important genomic regions for inter-individual and population-related variations in disease risk, especially for infectious and autoimmune diseases. This significance is highlighted by hundreds of notable associations identified through genome-wide association studies (GWAS) (Kennedy et al. 2017). HLA molecules are also crucial in directing and shaping the repertoire of T cell receptors (TCRs) during T cell maturation in the thymus, a process known as MHC restriction of TCRs. This process ensures that TCRs do not recognize HLA-presenting self-antigens, thereby promoting tolerance and preventing autoimmune responses. Classical class Ia HLA genes are broadly expressed on nucleated cells, allowing CD8+ T cells to recognize and eliminate cells infected with intracellular pathogens, such as viruses. In contrast, classical class IIa genes are expressed only on antigen-presenting cells (dendritic cells, B lymphocytes, and macrophages), presenting antigens to CD4+ T cells, which activate immune responses to target extracellular pathogens. Non-classical class Ib HLA molecules, primarily found in immune and endothelial cells, modulate immune responses by interacting with specific activating or inhibitory receptors; class IIb molecules, expressed on professional antigen-presenting cells, are essential for selecting peptides subsequently presented by classical class IIa molecules, thus fine-tuning the immune response (Beltrami et al. 2023).

Multiple studies across vertebrate species indicate that pathogen-driven selection is a key mechanism in maintaining MHC diversity (Hill et al. 1991; Prugnolle et al. 2005; Sanchez-Mazas et al. 2017). It is widely believed that different forms of balancing selection such as heterozygote advantage, frequency-dependent selection (rare allele advantage), or selection varying in space and time, primarily maintain the polymorphism of MHC genes in humans and other species (Hedrick 2007; Solberg et al. 2008; Key et al. 2014). Furthermore, ancient balancing selection can result in trans-species polymorphism, where many alleles appear to be older than the species in which they are found (Klein et al. 1998). A characteristic of long-term balancing selection is high polymorphism and an excess of alleles with intermediate frequencies close to the balanced variant (Siewert and Voight 2017, 2020; Bitarello et al. 2023). In contrast to balancing selection, positive directional selection results in adaptively important genetic variants increasing in frequency, leading to fixation or near fixation, resulting in the occurrence of a selective sweep and reduced variability in the area near the selected locus (Hedrick 2007). While most studies support balancing selection as the main evolutionary mechanism maintaining diversity at MHC genes, there is also evidence suggesting that positive directional selection may operate on some MHC loci (Sanchez-Mazas et al. 2017; Meyer et al. 2018; Harris and DeGiorgio 2020; Caro-Consuegra et al. 2022).

In a recent study, we showed that the Maniq are closely related to other Semang populations on the Thai-Malay Peninsula and share their ancestry with the ancient Hòabìnhian hunter-gatherers of mainland Southeast Asia (Göllner et al. 2022). The Semang, traditionally hunter-gatherers also known for their nomadic lifestyle, are part of the Orang Asli groups, meaning “original people.” The rainforest and their subsistence strategies likely expose them to diverse pathogens. However, HLA diversity among Southeast Asian hunter-gatherers remains underexplored. To date, only a few studies have investigated HLA variation in Orang Asli (including few Semang) populations in Malaysia (Jinam et al. 2010, 2022; Tasnim et al. 2016), underscoring the need for further research in this region. Although we are not aware of any specific health data published for the Maniq, studies have shown that closely related Orang Asli groups in Malaysia exhibit high prevalence rates of various parasites and infectious diseases (Mahmud et al. 2022). Given their close genetic relationships and geographic proximity, it is reasonable to infer that the Maniq face similar pathogen pressures. In this study, we use whole-genome sequencing (WGS) data to investigate the immunogenetic landscape of the Maniq by examining HLA diversity and pinpointing signatures of balancing and recent positive selection.

## Material and methods

### Ethical considerations and sample collection

This study was approved by the Ethics Committee of the University of Vienna (reference no. 00444) and the Khon Kaen University Ethics Committee for Human Research (reference no. HE622223). Saliva samples were collected from Maniq individuals (*n* = 21) who provided informed consent, using the Oragene DNA (OG-500) collection kit (DNA Genotek Inc., Canada). All study participants identified themselves as members of the Maniq people and were at least 18 years old. We visited the Maniq people several times to explain this and our previous study (Göllner et al. 2022). The study was performed in accordance with the ethical standards as laid down in the 1964 Declaration of Helsinki and its later amendments.

### Whole-genome sequencing and variant calling

DNA isolation was previously performed as part of a prior study (Göllner et al. 2022; Herzog et al. 2025). In this study, whole-genome sequencing (WGS) with high coverage was conducted. The sequencing libraries were prepared using 100 ng of DNA and the TruSeq Nano DNA sample preparation kit (Illumina Inc.), incorporating unique dual indexes from Illumina. Library preparation followed the manufacturer’s guidelines, and next generation sequencing was carried out on the NovaSeq 6000 platform with an S4 flow cell and v1.5 chemistry, producing paired-end reads of 150 base pairs in length. The Genome Analysis Toolkit (GATK) pipeline’s best practices were employed for data pre-processing and variant calling (DePristo et al. 2011; Van der Auwera et al. 2013). We employed Variant Quality Score Recalibration (VQSR) with GATK recommended thresholds (truth sensitivity cutoff of 99.0% for single-nucleotide polymorphism (SNP) and 99.9% for indels). Additionally, variants were filtered for Hardy–Weinberg equilibrium (*p* < 1 × 10⁻⁶), and we assessed individual and variant-level missingness using standard QC measures (genotype call rates > 95%). The reads were aligned to the GRCh38 human reference genome using the “bwa mem” algorithm from the Burrows-Wheeler Aligner v0.7.17 (Li and Durbin 2010). Following alignment, duplicate reads were identified and marked with the MarkDuplicates tool, and base quality scores were recalibrated using the BaseRecalibrator and ApplyBQSR tools from GATK v4.1.4.1. Variants were initially called on a per-sample basis using HaplotypeCaller in GVCF mode (Poplin et al. 2018), and the individual GVCF files were then combined into a multi-sample gVCF using the CombineGVCFs tool. Joint genotyping was performed with GenotypeGVCFs, and variant filtering was performed with Variant Quality Score Recalibration, utilizing the VariantRecalibrator and ApplyRecalibration tools from GATK v4.1.4.1, resulting in a final multi-sample VCF file. To ensure unrelated samples, we used the R package SNPRelate (Zheng et al. 2012), first pruning SNPs in linkage disequilibrium (LD; ld.threshold = 0.5), then estimating identity-by-descent using a maximum likelihood approach (snpgdsIBDMLE, maf = 0.01, missing rate = 0.05). Applying a kinship cutoff of 0.125, we retained 12 unrelated Maniq individuals including a trio for downstream analyses.

### HLA genotyping from WGS data

To ensure high accuracy in HLA genotyping, we employed two recently published bioinformatic approaches — HLA-HD v1.7.0 (Kawaguchi et al. 2017) and T1 K v1.0.8 (Song et al. 2023) — which have both been validated for high accuracy in determining HLA alleles from WGS data (Dashti et al. 2024; Lai et al. 2024). Paired-end FASTQ data were analyzed to obtain HLA genotypes for class Ia (*A*, *B*, *C*), class IIa (*DRA*, *DRB1*, *DQA1*, *DQB1*, *DPA1*, *DPB1*), as well as non-classical class Ib (*E*, *F*, *G*) and class IIb (*DMA*, *DMB*, *DOA*, *DOB*) genes. HLA-HD also requires the software bowtie2 v2.5.4 (Langmead and Salzberg 2012), Samtools v1.2.0 (Danecek et al. 2021), and the Picard software (Picard Toolkit 2019. Broad Institute, release 3.2.0; https://broadinstitute.github.io/picard/) to filter reads and to extract mapped reads from the WGS data. T1 K first extracts candidate reads from the FASTQ files and computes the abundance of all the input alleles simultaneously using the weighted expectation–maximization (EM) algorithm to maximize the likelihood of read alignments to the reference HLA alleles. The reads were compared to a reference panel from the IPD-IMGT/HLA database Release 3.57 (Robinson et al. 2020).

### HLA allele-based analysis

We analyzed HLA allele and haplotype frequencies using PyPop v1.1.0 (Lancaster et al. 2024). Hardy–Weinberg proportions were evaluated with Guo and Thompson’s Monte Carlo exact test and neutrality with the Ewens–Watterson (EW) homozygosity test using Slatkin’s implementation. Following PyPop’s two-tailed test, *p* < 0.025 indicates balancing selection, whereas* p* > 0.975 suggests directional selection or drift. Population structure was explored with principal-component analysis (PCA) in R v4.4.0 (R Core Team 2024). Besides the Maniq, we included published HLA frequency datasets for two Malaysian Semang groups—Jehai (JEH) and Kintaq (KIN) (Jinam et al. 2010, 2022; Tasnim et al. 2016)—along with East Asian (EAS) populations from the 1000 Genomes Project (Supplementary Table 1), the Tao of Taiwan, Papuans (Goroka Asaro and Madang), Māori, and Aboriginal Australians (Kimberley and Cape York). Source frequencies were taken from the 1000 Genomes HLA panel (Gourraud et al. 2014) and the Allele Frequency Net Database (AFND; Gonzalez-Galarza et al. 2020). PCA was performed based on allele frequencies from five classical HLA loci (*HLA-A*, *HLA-B*, *HLA-C*, *HLA-DQB1*, and *HLA-DRB1*). For most populations, frequencies for all five loci were available; however, for the Tao population, *DQB1* data were not available, and PCA was conducted using the available loci only. Allele frequencies for each locus were pivoted to wide format (missing values set to zero), then centered and scaled prior to PCA via the prcomp function (base R stats package). Principal components were merged with population metadata, and the proportion of variance explained by each PC was recorded. PCA results were visualized using ggplot2 (Wickham 2016), plotting PC1 against PC2.

### SNP-based analyses in the MHC region

For the subsequent natural selection and population differentiation analyses, we focused on single-nucleotide polymorphism (SNP) genotype data from chromosome 6, covering the entire MHC region.

### Quality control, phasing, and annotation

Variants were filtered to remove multi-allelic sites and those deviating from Hardy–Weinberg equilibrium (HWE) (*p* < 1 × 10^−6^) using BCFtools v1.2.0 and VCFtools v0.1.16 (Danecek et al. 2011, 2021). Genotypes were phased with SHAPEIT5 (Hofmeister et al. 2023) using the *phase_common* algorithm for unrelated samples with default parameters. Genetic maps aligned to the GRCh38 reference genome were obtained from the SHAPEIT repository (https://github.com/odelaneau/shapeit4/tree/master/maps). Phasing utilized an Asian genetic ancestry reference panel (Supplementary Table 1) created from pre-phased, high-coverage (30 ×) genotype data from the 1000 Genomes Project (GRCh38) (Byrska-Bishop et al. 2022). We used Ensembl Variant Effect Predictor (McLaren et al. 2016) to annotate SNPs with gene symbols, biotypes, and consequence types based on GRCh38.p14. Additionally, expression quantitative trait loci (eQTLs) were accessed from GTEx Portal V8 (dbGaP Accession phs000424.v8.p2) (Ardlie et al. 2015) to investigate whether SNPs associated with selected HLA genes function as eQTLs.

### Detection of positive selection

To detect positive selection, we applied the integrated haplotype score (iHS) (Voight et al. 2006) and cross-population extended haplotype homozygosity (xp-EHH) (Sabeti et al. 2007) methods on phased autosomal chromosomes, using selscan v1.2.0a (Szpiech and Hernandez 2014). The iHS method assesses extended haplotype homozygosity (EHH) around derived and ancestral alleles at candidate SNP sites, with alleles under positive selection showing unusually long-range linkage disequilibrium (LD) relative to allele frequency. Significant iHS values (≤ −2.0 for derived alleles; ≥ 2.0 for ancestral alleles) indicate positive selection. We calculated iHS in non-overlapping 100-kb windows, normalizing scores with 100 frequency bins across the genome. To compare selection between populations, we used xp-EHH, which examines EHH decay differences at a locus between the test and reference populations. A positive xp-EHH score (> 2.0) suggests stronger selection in the test population. We used the Kinh in Ho Chi Minh City (KHV) population from the 1000 Genomes Project as the reference population, with unstandardized xp-EHH scores normalized using default settings. SNPs in the 99.9 th percentile (absolute iHS > 2.9 and xp-EHH > 2.4) were considered candidates under positive selection.

### Detection of balancing selection

We used the BetaScan software (Siewert and Voight 2017) to detect long-term signatures of balancing selection in the MHC region. We employed the BetaScan2 algorithm to calculate standardized Beta2 scores (Beta2_std) (Siewert and Voight 2020), leveraging chimpanzee as an outgroup to infer ancestral alleles. Ancestral allele data were obtained from the all.epo.gz file derived from the Enredo-Pecan-Ortheus (EPO) multi-species alignments, as used in the original BetaScan studies (Siewert and Voight 2017, 2020). This file was downloaded from the BetaScan GitHub repository and is based on alignments to the GRCh37/hg19 reference genome. Since the EPO ancestral alignments are aligned to the GRCh37/hg19 reference genome, we first converted the VCF files to GRCh37 coordinates using Picard’s LiftoverVcf tool. We obtained the necessary chain file for liftover (hg38 ToHg19.over.chain) and the GRCh37 human reference genome from the UCSC Genome Browser (Raney et al. 2024). We used glactools (Renaud 2018) to convert phased SNP genotype data from chromosome 6 into the folded site frequency spectrum format required for BetaScan analysis. SNPs with Beta2_std scores above the 99.9 th percentile (Beta2_std > 17.23) were considered outlier loci and thus putative candidates under balancing selection. Although newer EPO alignments are available on GRCh38, we followed the original BetaScan2 setup to maintain consistency with prior studies and ancestral state inference pipelines.

### FST analysis

The phased Maniq SNP genotype data were merged with the phased, high-coverage genotype data (aligned to GRCh38) from the 1000 Genomes Project from the study (Byrska-Bishop et al. 2022), consisting of the four super-populations with East Asian (EAS), South Asian (SAS), European (EUR) and African (AFR) genetic ancestry. We calculated genome-wide pairwise (Maniq vs. 1000 Genomes super-populations) *FST* values for each variant using the Weir and Cockerham method (Weir and Cockerham 1984) implemented in VCFtools. Negative *FST* values were set to zero. We then computed global locus-specific *FST* values and standard deviations (sd) in R v4.4.0.

## Results

### Maniq HLA allele and haplotype diversity

The average sequencing depth for chromosome 6 was 28.3 ×, ranging from 19.52 to 40.07 ×. We employed two methods, HLA-HD (Kawaguchi et al. 2017) and T1K (Song et al. 2023), to identify HLA alleles in the Maniq population. Both methods identified the same HLA alleles. In total, we detected 32 HLA alleles across the class Ia loci (*HLA-A*, *HLA-B*, *HLA-C*) and class IIa loci (*HLA-DRA*, *HLA-DRB1*, *HLA-DQA1*, *HLA-DQB1*, *HLA-DPA1*, *HLA-DPB1*), as well as 14 alleles in the non-classical HLA loci, including class Ib genes (*HLA-E*, *HLA-F*, *HLA-G*) and class IIb genes (*HLA-DMA*, *HLA-DMB*, *HLA-DOA*, *HLA-DOB*) (Tables 1 and 2, respectively). Most of these alleles are also common in several Southeast Asian populations (in accordance with the HLA Allele Frequency Net Database, AFND) (Gonzalez-Galarza et al. 2020). The prevalence of these common alleles suggests shared ancestry with other Southeast Asian populations and potential local adaptation. A literature search indicated that several of these HLA alleles are associated with increased resistance or susceptibility to widespread human infectious diseases (Supplementary Table 2). However, some of the detected HLA alleles, such as B*27:06:01, B*38:02:01, and C*07:199:01, are rare in the broader Asian region (in accordance with AFND). All HLA loci were in Hardy–Weinberg equilibrium (HWE) (Supplementary Table 3). The Ewens–Watterson (EW) test, however, revealed significant deviations from neutrality for loci *DQA1* (*p* = 0.9859), *DMA* (*p* = 1.0000), and *DMB* (*p* = 1.0000), each showing greater observed homozygosity than expected (Supplementary Table 4). These findings suggest directional selection or genetic drift acting on these loci. All other loci conformed to neutral expectations. Table 3 presents the estimated HLA haplotype frequencies. The most common haplotypes in both HLA class I and class II loci are also found in closely related Semang populations, such as the Jehai and Kintaq, indicating most likely shared ancestry or similar pathogen-driven selection. Additionally, the AFND database shows that the most common HLA haplotypes occur at very low frequency and predominately in East Asian populations. The presence of these haplotypes at low frequencies suggests that the Maniq may have retained some ancestral haplotypes that are now less common elsewhere, possibly due to the effects of genetic drift and isolation.

**Table 1:** HLA class Ia and class IIa allele frequencies in the Maniq population (*n* = 12)

	**Allele**	**Frequency**
HLA class Ia gene		
* A*	A*24:07:01	0.7917
	A*02:01:02	0.1250
	A*11:01:01	0.0417
	A*24:02:01	0.0417
* C*	C*03:04:01	0.8333
	C*07:199:01	0.1250
	C*07:02:01	0.0417
* B*	B*13:01:01	0.7917
	B*18:01:01	0.1250
	B*27:06:01	0.0417
	B*38:02:01	0.0417
HLA class IIa gene		
* DRA*	DRA*01:01:01	0.9167
	DRA*01:02:02	0.0833
* DRB1*	DRB1*15:01:01	0.7917
	DRB1*09:01:02	0.1250
	DRB1*12:02:01	0.0417
	DRB1*15:02:01	0.0417
* DQA1*	DQA1*01:02:01	0.7917
	DQA1*03:02	0.0833
	DQA1*01:01:01	0.0417
	DQA1*03:01:01	0.0417
	DQA1*06:01:01	0.0417
* DQB1*	DQB1*05:02:01	0.7917
	DQB1*03:03:02	0.1250
	DQB1*03:01:01	0.0417
	DQB1*05:01:01	0.0417
* DPA1*	DPA1*01:03:01	0.8750
	DPA1*02:01:01	0.0833
	DPA1*02:02:02	0.0417
* DPB1*	DPB1*02:01:02	0.8750
	DPB1*13:01:01	0.0833
	DPB1*05:01:01	0.0417

**Table 2:** HLA class Ib and class IIb allele frequency in the Maniq population (*n* = 12)

	**Allele**	**Frequency**
HLA class Ib gene		
* E*	E*01:03:02	0.7917
	E*01:03:01	0.2083
* F*	F*01:01:01	1.0
* G*	G*01:04:01	0.7917
	G*01:01:01	0.1667
	G*01:01:03	0.0417
HLA class IIb gene		
* DMA*	DMA*01:01:01	0.9167
	DMA*01:02:01	0.0417
	DMA*01:03:01	0.0417
* DMB*	DMB*01:01:01	0.9583
	DMB*01:07:01	0.0417
* DOA*	DOA*01:01:02	0.8750
	DOA*01:01:04	0.1250
* DOB*	DOB*01:01:01	1.0

**Table 3:** HLA haplotype frequency estimates for class Ia and class IIb genes in the Maniq population

		**Frequency**
HLA class Ia	*A* ~ *C* ~ *B*	
	*24:07:01* ~ *03:04:01* ~ *13:01:01*	0.7474
	*02:01:01* ~ *07:199:01* ~ *18:01:01*	0.0807
	*02:01:01* ~ *03:04:01* ~ *13:01:01*	0.0443
	*24:07:01* ~ *07:199:01* ~ *18:01:01*	0.0443
	*11:01:01* ~ *03:04:01* ~ *27:06:01*	0.0417
	*24:02:01* ~ *07:02:01* ~ *38:02:01*	0.0417
HLA class IIa	*DRA* ~ *DRB1* ~ *DQA1* ~ *DQB1* ~ *DPA1* ~ *DPB1*	
	*01:01:01* ~ *15:01:01* ~ *01:02:01* ~ *05:02:01* ~ *01:03:01* ~ *02:01:02*	0.7917
	*01:01:01* ~ *09:01:02* ~ *03:02* ~ *03:03:02* ~ *01:03:01* ~ *02:01:02*	0.0833
	*01:02:02* ~ *12:02:01* ~ *06:01:01* ~ *03:01:01* ~ *02:01:01* ~ *13:01:01*	0.0417
	*01:01:01* ~ *09:01:02* ~ *03:01:01* ~ *03:03;02* ~ *02:02:02* ~ *05:01:01*	0.0417
	*01:02:02* ~ *15:02:01* ~ *01:01:01* ~ *05:01:01* ~ *02:01:01* ~ *13:01:01*	0.0417

### HLA diversity and population structure in the Semang

**Fig. 1:**
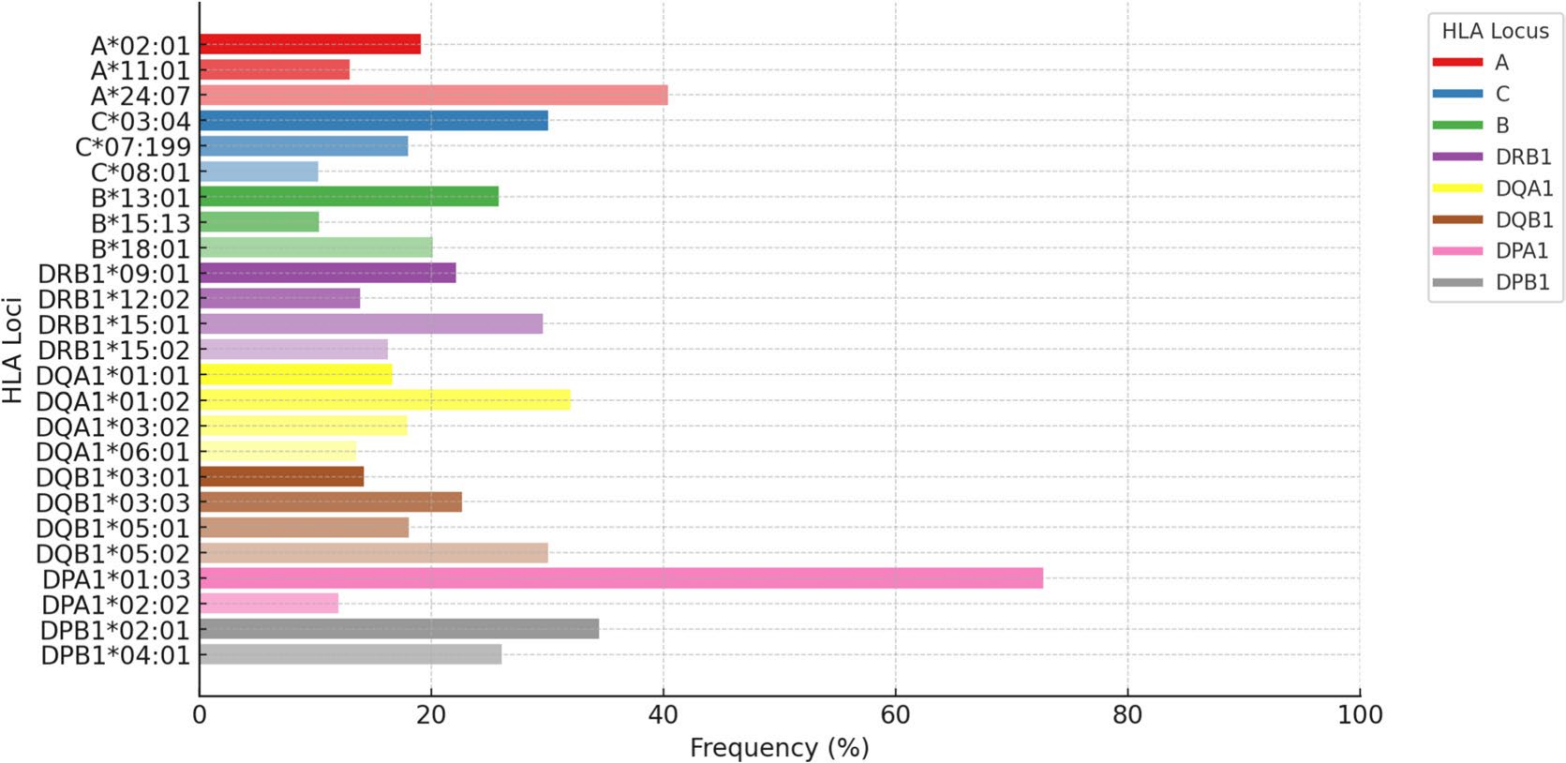
Most common HLA alleles (≥ 10%) across HLA loci in the Semang (*n* = 75)

**Fig. 2:**
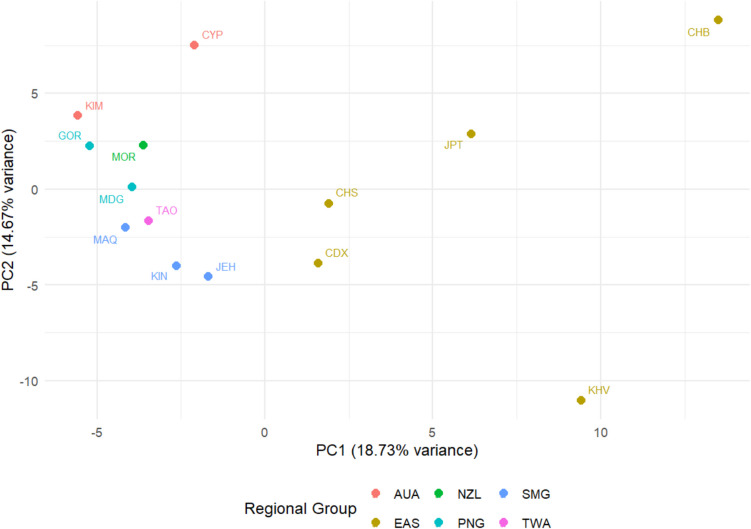
Principal component analysis (PCA) of HLA allele frequencies. PC1 (18.73% of the variance) is plotted against PC2 (14.67%). Point colors indicate regional groupings: Semang populations (SMG) from the Thai–Malay Peninsula—Maniq (MAQ), Jehai (JEH), and Kintaq (KIN); East Asian populations (EAS) from the 1000 Genomes Project (population codes provided in Supplementary Table 1); Aboriginal Australians (AUA) from Kimberley (KIM) and Cape York Peninsula (CYP); Māori (MOR) from New Zealand (NZL); Papuans from Goroka (GOR) and Madang (MAD) in Papua New Guinea (PNG); and the Tao (TAO) from Taiwan (TWA)

The most common HLA alleles found across Semang populations (combined Maniq, Jehai, and Kintaq) are shown in Fig. 1. To explore their population structure in a broader regional context, we performed principal component analysis (PCA) based on HLA allele frequencies, incorporating additional populations from East Asia (1000 Genomes Project), the Tao from Taiwan, Māori from New Zealand, Aboriginal Australians, and Papuans from Papua New Guinea. The first two principal components (PC1, 18.73%; PC2, 14.67%) captured major axes of differentiation, together explaining approximately one-third (33.4%) of total genetic variance (Fig. 2). Subsequent principal components explained progressively less variance (PC3, 12.46%; PC4, 10.54%; PC5, 7.86%), indicating diminishing contributions to overall population structure.

### MHC under recent positive selection in the Maniq

**Fig. 3:**
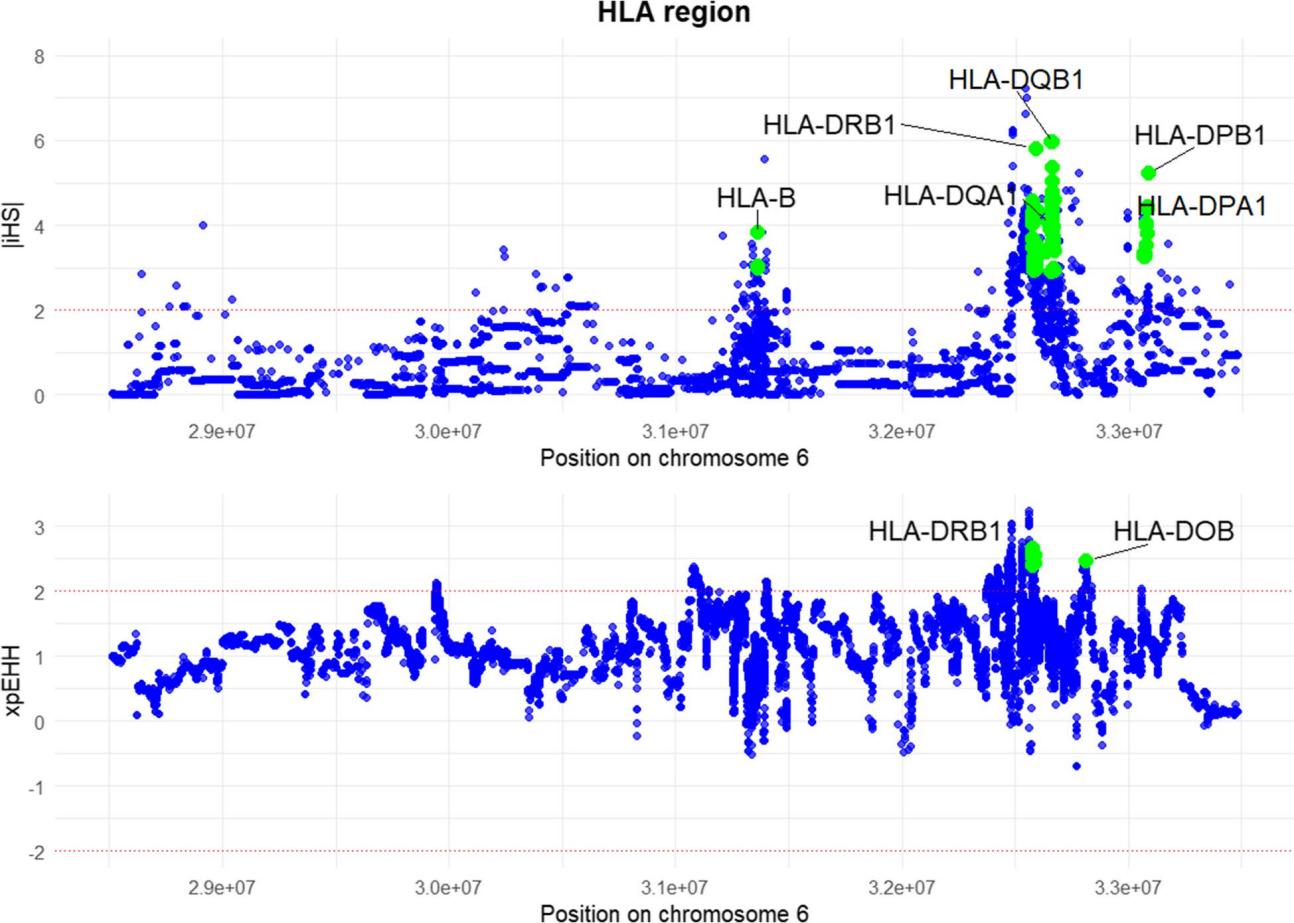
|iHS| and xp-EHH scores plotted across the HLA region. Red dotted line indicates significant iHS and xp-EHH scores > 2.0, and green dots highlight the outlier HLA loci with |iHS| values > 2.9 and xp-EHH > 2.4

The iHS analysis revealed that within the MHC, the *HLA-B* and all class IIa genes are candidates under recent positive selection. Additionally, the xp-EHH analysis identified *HLA-DRB1* and the non-classical class IIb locus *HLA-DOB* as candidates under positive selection (Fig. 3). Unlike classical HLA molecules, *HLA-DOB* is primarily expressed in lysosomes within B cells and plays a regulatory role in HLA-DM-mediated peptide loading onto HLA class II molecules. The lead SNPs with the highest iHS and xp-EHH values as well as locus-specific *F*_ST_ values are listed in Supplementary Tables 5 and 6, respectively. Notably, the SNPs under selection are functional variants that serve as eQTLs, potentially modulating HLA gene expression and influencing immune responses. Additionally, the positively selected SNPs at *DPA1* and *DPB1* exhibit complete linkage disequilibrium with two 3′UTR variants: rs3077 and rs9277535, respectively. These variants act as strong cis-eQTLs in immunologically relevant tissues, including the liver, whole blood, and spleen, as shown in the GTEx database, and have been strongly associated with chronic hepatitis B virus (*HBV*) infection outcomes in Asian populations (Kamatani et al. 2009; An et al. 2011; Ou et al. 2019, 2021). This suggests that pathogen-driven selective pressures — particularly from *HBV*—may have played a key role in shaping HLA diversity in the Maniq population.

### MHC loci under balancing selection in the Maniq

**Fig. 4:**
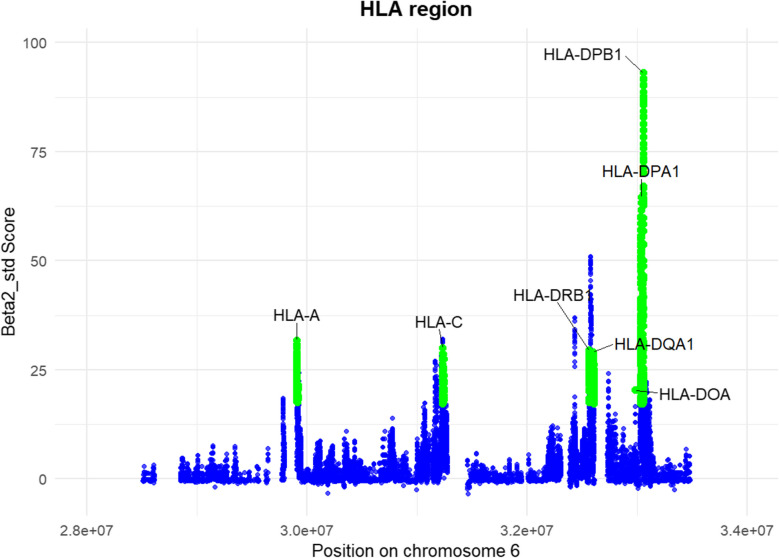
Standardized Beta2 (Beta2_std) scores plotted across the Maniq HLA region. Green dots highlight outlier HLA loci with Beta2_std scores > 17.23

Our analysis identified the MHC region as a distinct outlier (Supplementary Fig. 1), with several HLA loci showing strong evidence of long-term balancing selection, marked by high Beta2_std scores (Fig. 4). The SNPs with the highest Beta2_std scores, all of which also function as expression eQTLs, are given in Supplementary Table 7. This suggests that these variants not only play a role in genetic diversity but may also influence gene expression, further underlining their functional importance. The HLA genes *DPA1* and *DPB1* exhibit the highest Beta2_std scores, positioning them as major candidates for balancing selection in the Maniq population. In addition to the classical loci, we also detected the non-classical gene *DOA* as an outlier.

## Discussion

Our study presents the first analysis of HLA diversity in the Maniq, a small, isolated nomadic hunter-gatherer group inhabiting the rainforests of Southern Thailand. Recent mitochondrial and genome-wide studies (Kutanan et al. 2018; Göllner et al. 2022) have shed light on the Maniq’s demographic history, providing important context for interpreting their HLA diversity. Their unique demographic trajectory is characterized by early divergence from other Southeast Asian groups, limited gene flow, and prolonged isolation. Genome-wide data suggest that the Maniq retain substantial ancient (hunter-gatherer) Hòabìnhian-related ancestry, combined with approximately 35% East Asian–related admixture introduced through more recent contact with agriculturalist populations, followed by strong genetic drift and endogamy (Göllner et al. 2022). Such a demographic history has probably contributed to the reduced HLA diversity and the skewed allele-frequency patterns we observe. By identifying 32 alleles from classical HLA genes and 14 from non-classical HLA genes, we reveal patterns of genetic diversity shaped by demographic history, drift, and selection pressures.

We determined the HLA alleles using two recently developed methods, HLA-HD (Kawaguchi et al. 2017) and T1K (Song et al. 2023), applied to WGS data. Both methods yielded highly consistent results, underscoring the robustness of these methods in determining HLA diversity from WGS data. Notably, a few alleles at each HLA locus occur at very high frequencies in the Maniq population. This pattern mirrors findings from the Aché, a small Amerindian population of semi-nomadic hunter-gatherers in Eastern Paraguay (Tsuneto et al. 2003; Single et al. 2020). These parallels suggest that restricted HLA diversity, with a concentration of dominant alleles, may characterize very small, isolated indigenous populations, driven by genetic drift and local adaptation. Our previous research (Göllner et al. 2022) showed that the Maniq have one of the highest levels of genetic drift among living human populations, likely due to their prolonged geographic isolation, small population size, and history of endogamy. Consequently, HLA diversity in the Maniq is also relatively low compared to other Southeast Asian populations, possibly resulting in skewed HLA allele frequencies.

However, despite reduced diversity, the Maniq share specific HLA alleles with other Southeast Asian populations, indicating shared ancestry and potential common adaptive responses to regional pathogens. Some of these shared alleles have been found to be associated with protective immunity in some Asian populations (Supplementary Table 2). The most common class Ia alleles in the Maniq were A*24:07:01, B*13:01:01, and C*03:04:01; common class IIb alleles included DRB1*15:01:01, DQA1*01:02:01, DQB1*05:02:01, DPA1*01:03:01, and DPB1*02:01:02. The presence of rare HLA alleles such as B*27:06:01, B*38:02:01, and C*07:199:01, which are uncommon in the broader Asian region, suggests unique evolutionary pressures on the Maniq or the retention of ancestral alleles possibly lost in other populations. Principal component analysis (PCA) of HLA allele frequencies (Fig. 2) revealed distinct patterns of immunogenetic structure among Southeast Asian, Australian, and Oceanian populations. The first two principal components (PC1 and PC2) explained 18.7% and 14.7% of the total variance, respectively. The hunter-gatherer (Semang) populations Maniq, Jehai, and Kintaq formed a tight cluster, closely aligned with the Tao, an indigenous Austronesian-speaking group of Taiwan, and the indigenous groups from Papua New Guinea (Goroka, Madang). Interestingly, despite linguistic and cultural differences—the Semang being Austroasiatic speakers and the Tao being Austronesian—their clustering may reflect shared immune pressures from similar environments or ancient genetic links across Island and Mainland Southeast Asia (Jinam et al. 2012). In contrast, East Asian populations (Chinese, Japanese, Vietnamese) clustered distinctly, with Aboriginal Australians (Kimberly, Cape York) and Māori forming more differentiated positions along both PCs. This structure mirrors broader continental-scale HLA differentiation patterns, such as those identified by Arrieta-Bolaños et al. (2023), who reported marked HLA discontinuities across Southeast Asia, including along the Wallace Line. These findings suggest that populations, even if geographically isolated, are part of larger immunogenetic ecosystems shaped by migration, drift, and region-specific pathogen pressures.

Some of the HLA alleles such as C*07:199:01 are fairly common (~ 18%) across Semang groups, suggesting not only shared ancestry but potentially similar selection pressure maintaining specific HLA alleles at high frequency in the hunter-gatherers on the Thai-Malay Peninsula (Fig. 1). Notably, HLA-C*07:199:01 is nearly identical in sequence to C*07:04:01 (sequence data from the IPD-IMGT/HLA database (Robinson et al. 2020)), differing only at codon 95 in exon 3, where a phenylalanine to leucine substitution occurs. Due to this subtle difference, earlier studies based on lower-resolution HLA typing may have misclassified or failed to report the allele C*07:199 in Southeast Asian populations.

Although specific health data for the Maniq are lacking, studies on related Orang Asli groups in Malaysia indicate high infectious disease burdens, including infections with soil-transmitted helminths, protozoan parasites, and viral pathogens such as hepatitis B virus (*HBV*) (Sahlan et al. 2019; Mahmud et al. 2022). Notably, recent research has shown that *HBV* infection rates in some Semang populations in Malaysia are almost three times higher than the national average (Sahlan et al. 2019). Given their geographic proximity and similar subsistence practices, it is plausible that the Maniq experiences comparable pathogen pressures, which may have influenced the selection of specific HLA alleles associated with immunity to these diseases. Several HLA alleles detected in the Maniq are associated with either protective effects or increased susceptibility to specific pathogens. For instance, *HLA*-DPB1*02:01 is linked to protection against chronic *HBV* infection (Nishida et al. 2014; Ou et al. 2021), while HLA-DPB1*05:01 and DQB1*05:02 are associated with increased susceptibility (Zhu et al. 2007; Ou et al. 2021). The role of HLA genes in *HBV* infection is further supported by the prevalence of the common allele HLA-B*13:01 in the Maniq population, which has been associated with enhanced clearance of hepatitis B surface antigen in Asian populations (Miao et al. 2013). Furthermore, a study revealed that DQB1*03:03, which is a common allele in the Maniq population, is associated with protection against *Helicobacter pylori* (*Hp*) infection in Asian populations (Wang et al. 2015). Moreover, high prevalence of amoebiasis, caused by *Entamoeba histolytica* infection, has been recorded among Orang Asli (Anuar et al. 2012), and in a study on Bangladeshi children, it has been found that the heterozygous haplotype DQB1*06:01–DRB1*15:01 had protective effects against this infection (Duggal et al. 2004). Although DQB1*06:01 was not detected in our study, DRB1*15:01 has the highest frequency among *DRB1* alleles in the Maniq. This allele has also been found to be associated with a protective role against leishmaniasis (Blackwell et al. 2020). Several of the HLA alleles commonly found in the Maniq, including HLA-C*03:04, DRB1*09:01, DRB1*15:01, and DQB1*05:02, are among globally frequent alleles, reinforcing their potential long-term adaptive value (Sanchez-Mazas et al. 2024). Moreover, Arrieta-Bolaños et al. (2023) identified strong genetic barriers in HLA diversity across Southeast Asia, notably along the Wallace Line, suggesting that even isolated populations such as the Maniq are embedded within larger immunogenetic ecosystems shaped by shared histories of migration, drift, and exposure to regional pathogen pressures. These observations show that the HLA profile of the hunter-gatherer groups on the Thai-Malay Peninsula (see PCA in Fig. 2), while unique, reflects broader patterns of selection acting on human populations across time and geography.

We did not find any significant deviation from HWE at the HLA loci (Supplementary Table 3). The absence of HWE deviations despite pathogen-associated alleles may reflect a long-standing equilibrium shaped by past selection events, suggesting we may be observing the genetic legacy of ancient host–pathogen interactions. However, the EW tests of selective neutrality revealed significant (*p* > 0.975) deviations at *DQA1*, *DMA*, and *DMB*, with higher observed homozygosity than expected under neutrality, indicating potential directional selection or the effects of strong genetic drift at these loci (Supplementary Table 4). For other HLA loci, no significant deviation from neutrality was detected, suggesting more neutral patterns of allele frequency distribution.

The MHC SNP-based analyses revealed variants linked to different HLA genes under both balancing selection and positive selection (Figs. 3 and 4; Supplementary Tables 5,6 and 7). Balancing selection plays a crucial role in maintaining genetic diversity at immune-related loci, allowing populations to respond to a diverse array of pathogens. Our analysis identified classical HLA class Ia and all classical class IIa loci as candidates under balancing selection. Notably, *DPA1* and *DPB1* exhibited the highest Beta2_std scores (Supplementary Table 7), indicating strong selective pressure to maintain diversity at these loci. Interestingly, the non-classical HLA class IIb gene *DOA* also emerged as a candidate under balancing selection. Beyond that, we observed signals of recent positive selection at multiple classical HLA loci, and xp-EHH pinpointed the non-classical locus *DOB* as under positive selection (Fig. 3). The fact that both classical and non-classical HLA loci show different selective signatures underscores that the entire MHC region may experience varied evolutionary pressures, reflecting the broad pathogen landscape confronting the Maniq. Moreover, the overlapping evidence for long-term balancing and recent positive selection at certain HLA loci highlights a complex interplay wherein populations retain genetic diversity to combat numerous pathogens while also adapting to specific, high-prevalence threats. Notably, *DPA1* and *DPB1* under positive selection in the Maniq have also been reported as positively selected in indigenous Peruvian and Mesoamerican populations (Caro-Consuegra et al. 2022; Garcia et al. 2023). The lead SNPs at *DPA*1 and *DPB1* are in complete LD with functional 3′UTR variants (rs3077 and rs9277535) associated with *HBV* infection outcomes in Asian populations (Kamatani et al. 2009; An et al. 2011; Nishida et al. 2014; Mardian et al. 2017). A recent study reported higher levels of *HBV* diversity in Eastern Eurasia compared to Western Eurasia between 5000 and 3000 years ago, as well as a possible transition from non-recombinant *HBV* sub-genotypes to recombinant sub-genotypes (Sun et al. 2024). These historical patterns support the hypothesis that *HBV* may have exerted strong selective pressure favoring protective *HLA-DP* variants in the Maniq, evidenced by their elevated frequencies and high *F*_ST_ at these SNPs (Supplementary Fig. 2) relative to East Asian populations. These results indicate a central role of HLA genes in host–pathogen co-evolution and the adaptive immune response to viral pathogens like *HBV*.

In conclusion, HLA diversity in the Maniq reflects a dynamic interplay of genetic drift, balancing selection, and recent positive selection—shaped by unique demographic history and pathogen pressures. These insights underscore the adaptive importance of HLA alleles in small, isolated populations that face diverse pathogenic challenges. The high concordance between FASTQ- and SNP-based genotypes lends confidence to our findings, yet the small sample and the mapping pitfalls of short-read data caution against overinterpretation of locus-specific signals. Future work should combine (i) larger Maniq and neighboring Semang cohorts, (ii) long-read or graph-based assemblies to resolve the complex MHC, and (iii) matched immunological phenotypes. Such integrative studies will clarify how drift, migration, and region-specific pathogens have jointly sculpted HLA evolution in Southeast Asia’s remaining hunter-gatherer populations.

## Supplementary Information

Below is the link to the electronic supplementary material.

ESM 1 (DOCX 265 KB)

## Data Availability

HLA data used for comparative analyses were obtained from publicly available datasets, including previously published studies and the 1000 Genomes Project and from the Allele Frequency Net Database (https://www.allelefrequencies.net). However, due to legal and ethical restrictions, the whole-genome sequencing (WGS) data generated for the Maniq population cannot be made publicly available. Specific details regarding the datasets and sources used in this study are provided within the manuscript and supplementary materials.
