# Supplementary material for: HLA diversity and signatures of selection in the Maniq, a nomadic hunter-gatherer population in Southern Thailand
Source: Immunogenetics. 2025 Jun 9;77(1):23. doi: 10.1007/s00251-025-01380-0 (PMC12148999; doi:10.1007/s00251-025-01380-0)

**Supplementary Information**

**Supplementary Table 1.** Populations from the 1000 Genomes Project included in the study (<https://ftp.1000genomes.ebi.ac.uk/vol1/ftp/data_collections/1000G_2504_high_coverage/>).

| **Number of individuals** | **Population code** | **Population**  **description** | **Super population** | **Super population code** |
| --- | --- | --- | --- | --- |
| 99 | LWK | Luhya in Webuye, Kenya | Africa | AFR |
| 113 | GWD | Gambians from The Gambia | Africa | AFR |
| 99 | ESN | Esan in Nigeria | Africa | AFR |
| 108 | YRI | Yoruba in Ibadan, Nigeria | Africa | AFR |
| 91 | GBR | British in England and Scotland | Europe | EUR |
| 99 | FIN | Finnish in Finland | Europe | EUR |
| 107 | TSI | Toscani in Italia | Europe | EUR |
| 107 | IBS | Iberian populations in Spain | Europe | EUR |
| 86 | BEB | Bengali from Bangladesh | South Asian | SAS |
| 102 | ITU | Indian Telugu from the UK | South Asian | SAS |
| 96 | PJL | Punjabi from Lahore, Pakistan | South Asian | SAS |
| 103 | GIH | Gujarati Indians in Houston, Texas, USA | South Asian | SAS |
| 103 | CHB | Han Chinese in Beijing, China | East Asian | EAS |
| 104 | JPT | Japanese in Tokyo, Japan | East Asian | EAS |
| 99 | KHV | Kinhin Ho Chi Minh City, Vietnam | East Asian | EAS |
| 93 | CDX | Chinese Dai in Xishuangbanna, China | East Asian | EAS |

**Supplementary Table 2.** HLA alleles of class I (*A, C, B*) and class II (*DRB1, DQA1, DQB1, DPA1, DPB1*) genes identified in the Maniq population and their reported associations (reported OR = odds ratio and *p*-values) with common human infectious diseases in human populations.

| **HLA allele** | **Association with prevalent infectious diseases** | **OR** | ***p*** |
| --- | --- | --- | --- |
| *HLA-A*02:01* | *HCV* spontaneous clearance [1] | 1.839 | 0.004 |
| *HLA-A*11:01* | restricted CD8+ immunity against influenza [2] | - | - |
| *HLA-A*24:02* | susceptibility to dengue hemorrhagic fever [3] | 1.39 | <0.05 |
| *HLA-A*24:07* | – |  |  |
|  |  |  |  |
| *HLA-C*03:04* | may have a protective role in *HIV* infection [4] | - | - |
| *HLA-C*07:02* | – | - | - |
| *HLA-C*07:199* | – | - | - |
|  |  |  |  |
| *HLA-B*13:01* | increased clearance of *HBV* surface antigen [5] | 2.62 | 0.0004 |
| *HLA-B*18:01* | susceptibility to tuberculosis [6] | - | < 0.001 |
| *HLA-B*27:06* | potentially a protective role *HIV*, *HCV* [7, 8] | - | - |
| *HLA-B*38:02* | susceptibility to *cryptosporidium*, *astrovirus* infection [9] | 2.5,  2.3 | 0.0056,  0.0074 |
|  |  |  |  |
| *HLA-DRB1*09:01* | susceptibility to tuberculosis [10] | 1.82 | 0.0000988 |
| *HLA-DRB1*12:02* | protective against leprosy [11] | 0.69 | 0.000259 |
| *HLA-DRB1*15:01* | protective against leishmaniasis [12, 13]; strong association with multiple sclerosis [14] | 0.79, 0.63,  3.1 | 0.0003, 1.15×10⁻¹³,  1×10⁻³²⁰ |
| *HLA-DRB1*15:02* | protective against leishmaniasis [12] | 0.79 | 0.0084 |
|  |  |  |  |
| *HLA-DQA1*01:01* | – | - | - |
| *HLA-DQA1*01:02* | protection from chronic *HBV* infection liver cirrhosis and hepatocellular carcinoma in Chinese [15] | 0.39 | 0.003 |
| *HLA-DQA1*03:01* | – | - | - |
| *HLA-DQA1*03:02* | – | - | - |
| *HLA-DQA1*06:01* | protective role in tuberculosis [16] | 0.4 | 0.02 |
|  |  |  |  |
| *HLA-DQB1*03:01* | protective role in tuberculosis [16]; *HCV* spontaneous clearance [17] | 0.4,  3.899 | 0.01,  0.0017 |
| *HLA-DQB1*03:03* | protective against *Hp* infection [18] | 0.54 | 0.006 |
| *HLA-DQB1*05:01* | protective against *HBV* infection [19] | 0.59 | < 0.00001 |
| *HLA-DQB1*05:02* | increased risk of tuberculosis [16]; associated with chronic *HBV* infection [19]; susceptibility to *HBV* infection [20] | 2.6,  1.22,  18 | 0.01,  0.01,  <0.05 |
|  |  |  |  |
| *HLA-DPA1*01:03* | protection against *HBV* infection [21] | 0.51 | 3.15×10⁻¹⁰ |
| *HLA-DPA1*02:01* | susceptibility to *HBV* infection [21] | 1.88 | 2.61×10⁻^7^ |
| *HLA-DPA1*02:02* | susceptibility to *HBV* infection [21] | 1.89 | 2.66×10⁻^5^ |
|  |  |  |  |
| *HLA-DPB1*02:01* | protection against *HBV* infection [21, 22] | 0.68, 0.73 | 5.22×10^-6^, <0.0001 |
| *HLA-DPB1*05:01* | susceptibility to *HBV* infection [21, 22] | 1.45, 1.41 | 1.51×10^-4^, <0.001 |
| *HLA-DPB1*13:01* | protective against severe *SARS-CoV2* [23] | 0.49 | 0.019 |

**Abbreviations:** *HCV* (*hepatitis C virus*), *HBV* (*hepatitis B virus*), *EBV* (*Epstein-Barr Virus*), *SARS-CoV-2* (*Severe Acute Respiratory Syndrome Coronavirus type 2*), *HIV* (*Human Immunodeficiency Viruses*), *Hp* (*Helicobacter pylori*).

**References:**

[1] Huang J, Huang K, Xu R, et al. (2016) The Associations of HLA-A*02:01 and DRB1*11:01 with Hepatitis C Virus Spontaneous Clearance Are Independent of IL28B in the Chinese Population. Sci Rep. 6:31485.

[2] Habel JR, Nguyen At, Rowntree LC, et al (2022) HLA-A*11:01-restricted CD8+ T cell immunity against influenza A and influenza B viruses in Indigenous and non-Indigenous people. PLOS Pathogens 18(3):e1010337.

[3] Cook AC, Thibaut D, Pettersen T (2022) Major Histocompatibility Complex Class I and Dengue Hemorrhagic Fever: A Meta-Analysis of Human Leukocyte Antigens A*24 and B*44. Cureus 14(11):e31485.

[4] Ziegler MC, Nelde A, Weber JK, et al (2020) HIV-1 induced changes in HLA-C∗03 : 04-presented peptide repertoires lead to reduced engagement of inhibitory natural killer cell receptors. AIDS 34(12):1713-1723.

[5] Miao F, Sun H, Pan N, et al (2013) Association of human leukocyte antigen class I polymorphism with spontaneous clearance of hepatitis B surface antigen in Qidong Han population. Clin Dev Immunol 2013:145725.

[6] Kone A, Diarra B, Cohen K, et al (2019) Differential HLA allele frequency in Mycobacterium africanum vs Mycobacterium tuberculosis in Mali. HLA, 93:24-31.

[7] Goulder PJR., Watkins DI (2008) Impact of MHC class I diversity on immune control of immunodeficiency virus replication. Nature Reviews Immunology 8:619-630.

[8] McKiernan SM, Hagan R, Curry M, et al (2004) Distinct MHC class I and II alleles are associated with hepatitis C viral clearance, originating from a single source. Hepatology, 40(1):108-14.

[9] McCowin S, Moreau G, Haque R, et al (2021) HLA class I and II associations with common enteric pathogens in the first year of life. EBioMedicine, 67:103346.

[10] Toyo-Oka L, Mahasirimongkol S, Yanai H, et al (2017) Strain-based HLA association analysis identified HLA-DRB1*09:01 associated with modern strain tuberculosis, HLA, 90:149-156.

[11] Dallmann-Sauer M, Fava V, Gzara C, et al (2020) The complex pattern of genetic associations of leprosy with HLA class I and class II alleles can be reduced to four amino acid positions. PLOS Pathogens, 16(8):e1008818.

[12] Singh T, Fakiola M, Oommen J, et al (2018) Epitope-Binding Characteristics for Risk versus Protective DRB1 Alleles for Visceral Leishmaniasis. J Immunol. 200(8):2727-2737.

[13] Blackwell JM, Fakiola M, Castellucci LC (2020) Human genetics of leishmania infections. Hum Genet 139:813–819.

[14] The International Multiple Sclerosis Genetics Consortium & The Wellcome Trust Case Control Consortium 2, Sawcer S, Hellenthal G, et al (2011) Genetic risk and a primary role for cell-mediated immune mechanisms in multiple sclerosis. Nature 476(7359):214-9.

[15] Liu C, Cheng B (2007) Association of polymorphisms of human leucocyte antigen-DQA1 and DQB1 alleles with chronic hepatitis B virus infection, liver cirrhosis and hepatocellular carcinoma in Chinese. Int J Immunogenet. 34(5):373-8.

[16] Vejbaesya S, Chierakul N, Luangtrakool K, et al (2002) Associations of HLA class II alleles with pulmonary tuberculosis in Thais. Eur J Immunogenet 29:431-434.

[17] Huang J, Xu R, Wang M, et al (2019) Association of HLA-DQB1*03:01 and DRB1*11:01 with spontaneous clearance of hepatitis C virus in Chinese Li ethnicity, an ethnic group genetically distinct from Chinese Han ethnicity and infected with unique HCV subtype. J Med Virol. 91(10):1830-1836.

[18] Wang J, Zhang Q, Liu Y, et al (2015) Association between HLA-Ⅱgene polymorphism and Helicobacter pylori infection in Asian and European population: A meta-analysis. Microb Pathog 82:15–26.

[19] Ou G, Xu H, Yu H, et al (2018) The roles of HLA-DQB1 gene polymorphisms in hepatitis B virus infection. J Transl Med.16(1):362.

[20] Zhu XL, Du T, Li JH, et al (2007) Association of HLA-DQB1 gene polymorphisms with outcomes of HBV infection in Chinese Han population. Swiss Med Wkly, 137:114-120.

[21] Nishida N, Sawai H, Kashiwase K, et al (2014) New Susceptibility and Resistance HLA-DP Alleles to HBV-Related Diseases Identified by a Trans-Ethnic Association Study in Asia. PLOS ONE 9:e86449.

[22] Ou G, Liu X, Xu H, et al (2021) Variation and expression of HLA-DPB1 gene in HBV infection. Immunogenetics 73:253–261.

[23] Farias TDJ, Brugiapaglia S, Croci S, et al (2024) HLA-DPB1*13:01 associates with enhanced, and KIR2DS4*001 with diminished protection from developing severe COVID-19. HLA 103(1):e15251

**Supplementary Table 3.** Tests for deviation from Hardy–Weinberg Equilibrium (HWE) across classical and non-classical HLA loci in the Maniq population. HWE was assessed using Guo and Thompson’s exact Monte Carlo test (100,000 iterations). The number of observed and expected homozygotes are shown for each locus. No significant deviations from HWE were detected (*p* > 0.05 for all loci). Slatkin’s exact test *p*-values are also provided for comparison.

| **Locus** | **N** | | **k (alleles)** | **Homozygotes (Obs/Exp)** | **Guo & Thompson**  ***p*-value** | **Slatkin *p*-value** |
| --- | --- | --- | --- | --- | --- | --- |
| HLA-A | | 12 | 4 | 7/7.75 | 1 | 0.9170 |
| HLA-B | | 12 | 4 | 7/7.75 | 1 | 0.9170 |
| HLA-C | | 12 | 3 | 8/8.54 | 1 | 0.8102 |
| HLA-DRA | | 12 | 2 | 10/10.17 | 1 | 0.7193 |
| HLA-DRB1 | | 12 | 4 | 7/7.75 | 1 | 0.9170 |
| HLA-DQA1 | | 12 | 5 | 7/7.67 | 1 | 0.9859 |
| HLA-DQB1 | | 12 | 4 | 7/7.75 | 1 | 0.9170 |
| HLA-DPA1 | | 12 | 3 | 9/9.29 | 1 | 0.9069 |
| HLA-DPB1 | | 12 | 3 | 9/9.2 | 1 | 0.9069 |
| HLA-E | | 12 | 2 | 7/8.04 | 1 | 0.3867 |
| HLA-F | | 12 | 1 | 12/12 | - | - |
| HLA-G | | 12 | 3 | 9/7.88 | 0.4024 | 0.7277 |
| HLA-DMA | | 12 | 3 | 10/10.12 | 1 | 1.0000 |
| HLA-DMB | | 12 | 2 | 11/11.04 | 1 | 1.0000 |
| HLA-DOA | | 12 | 2 | 11/9.38 | 0.1292 | 0.5730 |
| HLA-DOB | | 12 | 1 | 12/12 | - | - |

**Supplementary Table 4.**  Slatkin's implementation of Ewens-Watterson (EW) homozygosity test of neutrality. Given are Observed F (Obs F), Expected F (Exp F), normalized deviate of the homozygosity F (Fnd) and the corresponding *p*-value. The column ‘Relation to F Neutral’ specifies whether the observed homozygosity is higher, lower, or about equal to the expected homozygosity. Entries marked with “–“ indicate loci that are monomorphic. Significant deviations from neutrality (directional selection or drift) are indicated by *p*-values > 0.975 (highlighted in bold).

| **HLA genes** | **Obs F** | **Exp F** | **Fnd** | ***p*-value of F** | **Relation to F Neutral** |
| --- | --- | --- | --- | --- | --- |
| **Classical HLA genes** |  |  |  |  |  |
| *HLA-A* | 0.6458 | 0.4602 | 1.4630 | 0.9170 | Higher |
| *HLA-C* | 0.7188 | 0.5765 | 0.9127 | 0.8102 | Higher |
| *HLA-B* | 0.6458 | 0.4602 | 1.4630 | 0.9170 | Higher |
| *HLA-DRA* | 0.8472 | 0.7441 | 0.6765 | 0.7193 | Higher |
| *HLA-DRB1* | 0.6458 | 0.4602 | 1.4630 | 0.9170 | Higher |
| *HLA-DQA1* | 0.6389 | 0.3759 | 2.4866 | **0.9859** | Higher |
| *HLA-DQB1* | 0.6458 | 0.4602 | 1.4630 | 0.9170 | Higher |
| *HLA-DPA1* | 0.7743 | 0.5765 | 1.3345 | 0.9096 | Higher |
| *HLA-DPB1* | 0.7743 | 0.5765 | 1.3345 | 0.9096 | Higher |
| **Non-classical HLA genes** |  |  |  |  |  |
| *HLA-E* | 0.6701 | 0.7441 | -0.4847 | 0.3867 | Lower |
| *HLA-F* | – | – | – | – | – |
| *HLA-G* | 0.6562 | 0.5765 | 0.5378 | 0.7277 | Higher |
| *HLA-DMA* | 0.8438 | 0.5765 | 1.8031 | **1.0** | Higher |
| *HLA-DMB* | 0.9201 | 0.7441 | 1.1547 | **1.0** | Higher |
| *HLA-DOA* | 0.7812 | 0.7441 | 0.2439 | 0.5730 | Higher |
| *HLA-DOB* | – | – | – | – | – |

**Supplementary Table 5.** HLA SNPs with the highest (|iHS| values and global locus-specific *F_ST_* values (standard deviation, sd) calculated across all analyzed populations. SNP functions were obtained from Ensembl Variant Effect Predictor (Assembly: GRCh38.p14) database and from GTEx v8.

| **Locus** | **SNP** | **Position** | **Function** | **\|iHS\|** | ***F_ST_*** (sd) |
| --- | --- | --- | --- | --- | --- |
| *B* | rs36057188 | 31360983 | upstream, eQTL | 3.8 | 0.878 (0.067) |
| *DRB1* | rs9270187 | 32588188 | intron, eQTL | 5.8 | 0.683 (0.034) |
| *DQA1* | rs9273067 | 32644436 | downstream, eQTL | 4.1 | 0.541 (0.143) |
| *DQB1* | rs1130432 | 32661352 | missense, eQTL | 5.4 | 0.507 (0.040) |
| *DPA1* | rs9380336 | 33072959 | intron, eQTL | 4.1 | 0.221 (0.182) |
| *DPB1* | rs9277516 | 33086503 | intron, eQTL | 5.3 | 0.236 (0.146) |

**Supplementary Table 6.** HLA SNPs with the highest xp-EHH values and *F_ST_* values (standard deviation, sd) calculated for the pairwise comparison Maniq versus East Asians. SNP functions were obtained from Ensembl Variant Effect Predictor (Assembly: GRCh38.p14) database and from GTEx v8.

| **Locus** | **SNP** | **Position** | **Function** | **xp-EHH** | ***F_ST_*** (sd) |
| --- | --- | --- | --- | --- | --- |
| *DRB1* | rs9269465 | 32574470 | downstream, eQTL | 2.7 | 0.240 (0.109) |
| *DOB* | rs12207915 | 32809975 | downstream, eQTL | 2.5 | 0.801 (0.147) |

**Supplementary Table 7. HLA** SNPs with top-scoring *Beta2_std* (*β^(2)^_std_*) scores and global locus-specific *F_ST_* values (sd = standard deviation) calculated across all analyzed populations. SNP functions were obtained from Ensembl Variant Effect Predictor (Assembly: GRCh37/hg19) database and from GTEx v8 – see for further information in Material and Methods.

| **Locus** | **SNP** | **Position** | **Function** | ***β^(2)^_std_*** | ***F_ST_*** (sd) |
| --- | --- | --- | --- | --- | --- |
| *A* | rs9260080 | 29909071 | 5’UTR, eQTL | 34.04 | 0.074 (0.068) |
| *C* | rs9264532 | 31234381 | downstream, eQTL | 30.07 | 0.062 (0.022) |
| *DRB1* | rs28366307 | 32560995 | upstream, eQTL | 29.53 | 0.032 (0.023) |
| *DQA1* | rs9272036 | 32598841 | intron, eQTL | 28.36 | 0.309 (0.077) |
| *DOA* | rs9276986 | 32980222 | upstream, eQTL | 20.35 | 0.219 (0.073) |
| *DPA1* | rs77527547 | 33034118 | intron, eQTL | 64.58 | 0.197 (0.181) |
| *DPB1* | rs9277511 | 33054215 | intron, eQTL | 93.08 | 0.201 (0.145) |


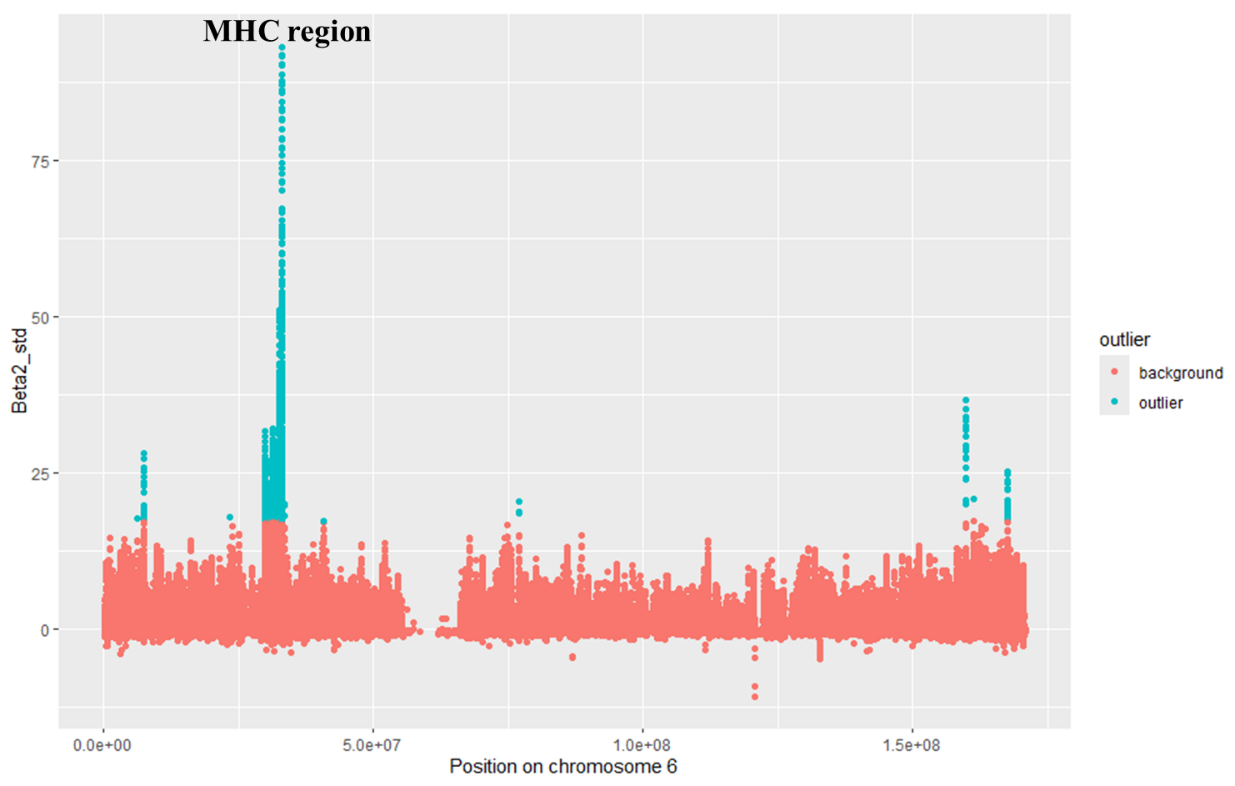
**Supplementary Figure 1.** Plot of Beta2_std scores across chromosome 6; the MHC region appears as distinct outlier region.

**Supplementary Figure 2. Allele frequencies of the two 3’UTR SNPs rs3077-G/A and rs9277535-A/G in the Maniq and East Asian populations (1000 Genomes Project).** These two variants are function as eQTLs in several tissues such as liver, whole blood, and spleen, and are in complete linkage disequilibrium (LD) with positively selected variants at the *HLA-DPA1* and *HLA-DPB1* loci in the Maniq population. The A alleles (shown in green) have been associated with a decreased risk of chronic *HBV* infection in Asian populations (e.g., Kamatani et al. 2009).


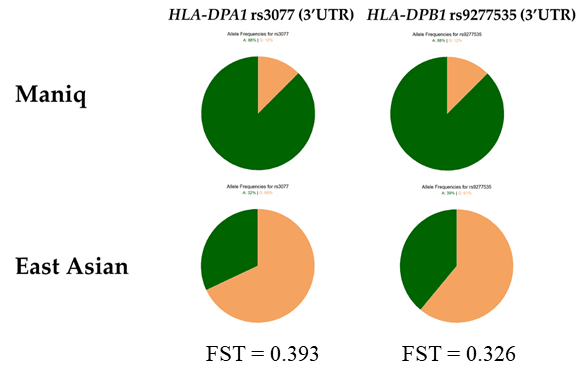

Supplement: Supplementary file 1 — (DOCX 265 KB) [file 251_2025_1380_MOESM1_ESM.docx]
